# Fungi and Mycotoxins in Feed Intended for Sows at Different Reproductive Stages in Argentina

**DOI:** 10.4061/2010/569108

**Published:** 2010-06-09

**Authors:** Carina Maricel Pereyra, Lilia Renée Cavaglieri, Stella Maris Chiacchiera, Ana María Dalcero

**Affiliations:** ^1^Departamento de Microbiología e Inmunología, Universidad Nacional de Río Cuarto, CONICET, Ruta 36, km 601, Río Cuarto, 5800 Córdoba, Argentina; ^2^Consejo Nacional de Investigaciones Científicas y Tecnológicas (CIC-CONICET), Carrera del Investigador Científico, Rivadavia 1917, CP C1033AAJ, Buenos Aires, Argentina; ^3^Departamento de Química, Universidad Nacional de Río Cuarto, Ruta 36, km 601, Río Cuarto, 5800 Córdoba, Argentina

## Abstract

The aim of this study was to evaluate fungi and contamination levels of aflatoxin B_1_, ochratoxin A, fumonisin B_1_, and zearalenone in raw materials and finished feed intended for sows at different reproductive stages. Total fungi, *Aspergillus*, *Penicillium*, and *Fusarium* species occurrence, were examined. *Aspergillus flavus*, *A. niger aggregate* spp., and *F. verticillioides* were the prevalent species. Fungal counts exceeded the levels proposed as feed hygienic quality limits (1 × 10^4^ colony forming units) at all reproductive stages. Aflatoxin B_1_, ochratoxin A, fumonisin B_1_, and zearalenone were detected by high-pressure liquid chromatography. Aflatoxin levels in 80% samples of finished sow feeds were over the permitted levels of 0.02 *μ*g g^−1^ (mean 228.2 ± 95 *μ*g Kg^−1^). Fumonisin B_1_ was detected in all tested raw materials at levels that varied from 50.3 to 1137.64 *μ*g Kg^−1^ and finished feed samples at levels that ranged from 99.8 to 512.4 *μ*g Kg^−1^. Aflatoxin B_1_, zearalenone, and ochratoxin A were not detected in raw materials. All finished feeds were negative for zearalenone contamination whereas all nonpregnant gilt samples were contaminated with low OTA levels (mean 0.259 ± 0.123). This fact requires periodic monitoring to prevent the occurrence of mycotoxicosis in animal production, to reduce the economic losses, and to minimize hazards to human health.

## 1. Introduction

Commercial mixed feeds are a basic element in modern animal production. They contain mixtures of home grown cereals and imported commodities among other ingredients. The use of such ingredients inevitably leads to the contamination of the final mixed feed with fungi [1]. Mycotoxins are fungal secondary metabolites associated with severe toxic effects to vertebrates and produced by important phytopathogenic spoilage fungi including *Aspergillus, Penicillium, Fusarium, *and *Alternaria *species [2]. 

Aflatoxins (AFs) are mycotoxins with high carcinogenic potential, especially in liver tissue, and possess an acute toxicity at high concentrations. Aflatoxins refer to a group of four mycotoxins, aflatoxin B_1_ (AFB_1_), aflatoxin B_2_ (AFB_2_), aflatoxin G_1_ (AFG_1_), and aflatoxin G_2_ (AFG_2_), and are mainly produced by *A. flavus* and *A. parasiticus* strains [2]. Ochratoxin A (OTA) is one of the most common and dangerous mycotoxins in foods and feeds produced mainly by *A. ochraceus*, *A. carbonarius,* and *A. niger* aggregate spp. in tropical regions, and *P. verrucosum* in temperate areas [3–5]. OTA has a potent toxicity and the nephrotoxic, hepatotoxic, teratogenic, carcinogenic, and immunosuppressive effects have been demonstrated in all mammalian species [6]. Fumonisin B_1_ (FB_1_), one of the most important mycotoxins is produced by *F. verticillioides* and associated with known outbreaks of porcine pulmonary edema [7]. Zearalenone (ZEA), an estrogenic metabolite mainly produced by *F. graminearum,* is the cause of hyperestrogenism, the estrogenic syndrome in pigs, and has been reported to occur not only in corn but also in other grains and silage in many areas of the world [6]. 

Continuous studies regarding the monitoring of these toxins in products to be used as pig feed are being performed in South America [8–10] and other countries [11]. However, no information is available regarding fungi and mycotoxins contamination in sow feed.

The aim of the present study was to determine the mycobiota and AFB_1_, OTA, FB_1_, and ZEA contamination from raw materials and finished feed intended for sows at different reproductive stages in Argentina.

## 2. Materials and Methods

### 2.1. Source of Samples and Feed Composition

Samples of sow feed were collected from two farms located in Baradero (latitude: 39°52′S; longitude: 59°29′W) Buenos Aires province, eastern region of Argentina. Feed was produced and processed on farm and was intended for reproductive sows. Ten samples (3 kg) of each raw material and finished reproduction sows feed were randomly obtained monthly from the line production and taken to the laboratory, from May to September 2008. Two kinds of raw materials, corn meal, and soybean intended for feed manufacturing, were collected. The composition of the three kinds of finished feed samples is described in Table 1. Primary samples were homogenized, milled, and quartered to get 1 kg laboratory samples. Mycological evaluation was immediately done. Then, all samples were stored at −20°C up to one week for mycotoxins analyses.

### 2.2. Mycological Determination

Total fungal counts from samples were performed onto dichloran rose bengal chloranphenicol agar (DRBC), a general media used for estimating total culturable fungi, onto dichloran 18% glycerol agar (DG18), a media that has low water activity (a_W_) and favours xerophilic fungi development [12]. Quantitative enumeration was done using the surface-spread method. Ten grams of each sample were homogenized in 90 mL 0.1% peptone water solution for 30 minutes in an orbital shaker. Serial dilutions (10^−2^ to 10^−3^) were made and 0.1 mL aliquots were inoculated in duplicates onto the culture media. Plates were incubated at 25°C for 7–10 days in darkness. Only plates containing 10–100 colony-forming units (CFUs) were used for counting. The results were expressed as CFU per gram of sample (CFU g^−1^). Representative colonies of *Aspergillus *and* Penicillium *spp. were transferred for subculturing to tubes containing malt extract agar (MEA) and *Fusarium *spp. were transferred to carnation leaf agar (CLA). Fungal species were identified according to taxonomic keys [12–14]. Results were expressed as isolation frequency (% of samples in which each genera was present) and relative density (% of isolation of each species among strains of the same genera).

### 2.3. Mycotoxins Analyses

Aflatoxin B_1_, OTA, FB_1_, and ZEA were determined in raw materials and finished reproductive sows feed as follows.

#### 2.3.1. Aflatoxin B_1_ Determination

A 50 g portion of sample was extracted with 150 mL methanol : water (80 : 20, v/v) during 60 minutes into a blend jar. The mixture was filtered through filter paper Whatman N°4 (Whatman, Inc., Clifton, New Jersey, USA) and a 2.5 mL aliquot taken and 2.5 mL of acetonitrile was added. This mixture was placed into 10 mL culture tube. Multifunctional columns Mycosep 228 (MFC, Romer Labs, Inc., MO., USA) were used for clean the samples. The extract was forced through frit, through 1-way valve, and through packing material. Purified extract (100 *μ*L) was collected in a column reservoir and diluted with 300 *μ*L of the mobile phase.

Aflatoxin B_1_ detection and quantification from each sample was performed by High-Performance Liquid Chromatography (HPLC) according to the methodology proposed by Trucksess et al. [15]. An aliquot (200 *μ*L) was derivatized with 700 *μ*L trifluoroacetic acid-acetic acid-water (20 : 10 : 70, v/v). Chromatographic separations were performed on a reversed phase column (Silica Gel, 150 × 4.6 mm id., 5-*μ* particle size, VARIAN, Inc. Palo Alto, USA). Methanol-water (60 : 40 v/v) was used as mobile phase at a flow rate 1 mL min^−1^. Fluorescence of AF derivatives was recorded at excitation and emission wavelengths of *λ* 360 nm and *λ* 460 nm, respectively. Standard curves were constructed with different levels of AFB_1_. This toxin was quantified by correlating peak heights of sample extracts with those of standard curves. The detection limit of the analytical method was 0.4 ng g^−1^.

#### 2.3.2. Ochratoxin A Determination

The detection of OTA in samples was performed by HPLC following the methodology proposed by Scudamore and MacDonald [16], with some modifications. Ground samples (50 g) were extracted with 100 mL of methanol : water (80 : 20, v/v). The mixture was shaken for 30 minutes and filtered. An aliquot of 10 mL was mixed with 40 mL of distilled water and filtered through a microfiber filter (VICAM, Digen Ltd., Exford). Ten mL of either portion was taken and transferred to an immunoaffinity column (Ochra-Test, Vicam, Digen Ltd. Oxford). The column was washed with 10 mL PBS containing 0.01% Tween 20 and then with 10 mL distilled water. Ochratoxin A was eluted from the column with methanol (HPLC grade), again at a flow rate of 1-2 drops per second. The eluate was evaporated to dryness in a stream of nitrogen. Prior to HPLC analysis, samples were redissolved in 200 *μ*L of the mobile phase. 

The HPLC apparatus used for determination of OTA was a Hewlett Packard chromatograph with a loop of 20 mL, equipped with a spectrofluorescence detector (excitation, *λ* 330 nm; emission, *λ* 460 nm) and a C18 column (Supelcosil LC-ABZ, Supelco; 150 mm, 4.6 mm, 5 mm particle size) connected to a precolumn (Supelguard LC-ABZ, Supelco; 20 mm, 4.6 mm, 5 mm particle size). The mobile phase was pumped at 1.0 mL min^−1^ and consisted of an isocratic system as follows: 57% acetonitrile, 41% water, and 2% acetic acid. Ochratoxin was quantified on the basis of HPLC fluorometric response compared with OTA standard (Sigma–Aldrich, St. Louis, MO, USA; purity >99%). The detection limit of the method was 0.1 ng g^−1^.

#### 2.3.3. Fumonisins Determination

Fumonisins content was determined by high-pressure liquid chromatography (HPLC). Samples (25 g) were grounded, extracted with 50 mL of methanol : water (30 : 10, v/v), and shaken 30 minutes in an orbital shaker. The extract was filtered through filter paper Whatman N°4. The extract was quantitatively analysed for FB_1_ and FB_2_ by HPLC using the methodology proposed by Shephard et al. [17] and modified by Doko et al. [18]. An aliquot (50 *μ*L) of this solution was derivatized with 200 *μ*L of *o-*phthaldialdehyde (OPA). The OPA solution was obtained by adding 5 mL of 0.1 M sodium tetraborate and 50 *μ*L of 2-mercaptoethanol to 1 mL of methanol containing 40 mg of OPA. Fumonisins OPA derivatives (20 *μ*L solution) were analysed using a reverse-phase high-pressure liquid chromatography/fluorescence detection system. The HPLC system consisted of a Hewlett Packard 1050 pump (Palo Alto, CA, USA) connected to a Hewlett Packard 3395 integrator. Chromatographic separations were performed on a stainless steel Supelcosil LC-ABZ, C 18 reverse-phase column (150 × 4.6 mm i.d., 5 *μ*m particle size; Supelco) methanol/0.1 M sodium dihydrogen phosphate. Fumonisins were quantified by using area measurements and by comparison with a reference standard solution. The standard solution was obtained by dissolving crystalline FB_1_ and FB_2_ (Division of Food Science and Technology, Pretoria, South Africa) in acetonitrile : water (10 : 10, v/v), at concentrations of 100 and 50 *μ*g mL^−1^, respectively. Detection limit of the method was 1 *μ*g Kg^−1^.

#### 2.3.4. Zearalenone Determination

Zeralenone analyses were performed by thin layer chromatography (TLC), following the methodology described in the Official Methods of Analysis [19]. Each sample (25 g) was extracted with 125 mL of methanol : water (60 : 40 v/v), 80 mL hexane, and 2 g NaCl and shaken 30 minutes in an orbital shaker. The mixture was filtered using filter paper Whatman N°4 (Whatman, Inc., Clifton, New Jersey, USA) and 25 mL of the filtrated methanol : water phase was extracted twice with 25 and 15 mL of chloroform, respectively. The chloroform phase was vacuum dried using a rotatory evaporator and the extract was redissolved in 200 *μ*L chloroform : acetone (9 : 1 v/v). The extract was screened by spotting 2 *μ*L, 5 *μ*L, and 10 *μ*L of each extract together with standard solution of toxin on a silica gel 60 TLC aluminium sheets (20 × 20 cm, Merck, Germany) and developed with chloroform : acetone (90 : 10 v/v). Chromatograms were air-dried and observed under 365 and 254 nm UV light. The relative amounts of ZEA were quantitatively determined by visual comparison under UV light with standard solutions of known toxin concentration. A detection limit of the used method was 50 *μ*g kg^−1^.

## 3. Results

Table 2 shows fungal counts from raw materials and finished sows feed from different culture media. Total fungal count analyses from raw materials show that corn obtained the highest values with means ranging from 1.5 × 10^4^ to 2.9 × 10^5^ CFU g^−1^ and 1 × 10^4^ to 1.5 × 10^5^ CFU g^−1^ in DRBC and DG18, respectively. Soybeans had counts that varied from <1 × 10^2^ to 3 × 10^3^ and 1 × 10^2^ to 1 × 10^3^ CFU g^−1^ from DRBC and DG18, respectively. All milled corn samples showed fungal contamination levels over 1 × 10^4^ CFU g^−1^, that this is the maximum recommended level [20]. In general, August and September had the highest fungal counts from both DRBC and DG18 media for milled maize.

All finished reproductive sow feed analysed samples had counts higher than 1 × 10^4^ CFU g^−1^. Means varied from 3.2 × 10^4^ to 8.2 × 10^4^ CFU g^−1^ in DRBC and from 2.4 × 10^4^ to 1.3 × 10^5^ CFU g^−1^ in DG18. September was the period with counts over 1 × 10^5^ CFU g^−1^ from both tested media, whereas sow samples were the most contaminated all over the sampling period.

**Figure 1:**
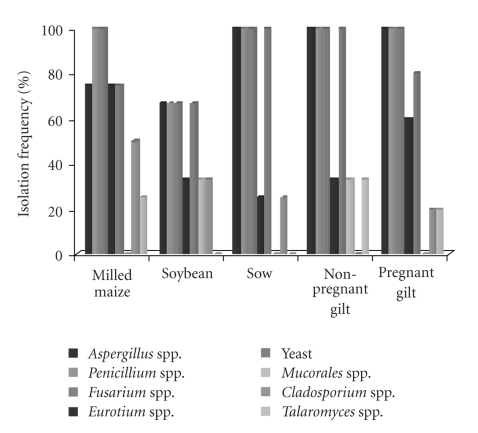
Isolation frequency of fungal genera (%) from raw materials and finished feed for sows at different reproductive stages.

Figure 1 shows the isolation frequency of fungal genera (%) from raw materials and finished reproduction sows feed. Milled maize and soybean samples contained at least one of the main mycotoxigenic genera. *Aspergillus* spp., *Penicillium* spp., *Fusarium* spp. and yeasts were isolated from all kinds of raw materials at frequencies that varied from 67% to 75%. *Eurotium* spp. and *Cladosporium* spp. contaminated from 75% to 50% samples of corn and 33% to 33% samples of soybean, respectively. Other fungal species such as Mucorales and *Talaromyces* spp. were isolated at low frequencies. All finished feed samples (100%) intended to reproduction sows samples (sow, Nonpregnant gilts, and pregnant gilts) were contaminated by *Aspergillus* spp., *Penicillium* spp., and *Fusarium* spp., Yeasts were isolated from all finished reproduction sows samples at levels that varied from 80% to 100%, whereas other fungal spp. such as Mucorales, *Cladosporium* spp., and *Talaromyces* spp. were present at low levels.

**Figure 2:**
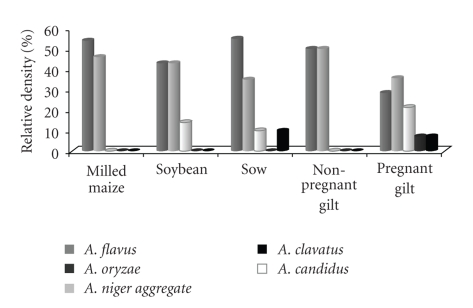
Relative density (%) of *Aspergillus* spp. isolated from raw materials and finished feed for sows at different reproductive stages.

Figure 2 shows the relative density of isolated* Aspergillus* spp. from raw materials and finished feed. *Aspergillus flavus* and *A. niger* aggregate spp. were isolated from all raw materials and finished feed. *A. flavus* was isolated at levels that ranged from 43% to 54% and from 35% to 55% for raw materials and finished feed, respectively. *Aspergillus niger* aggregate spp. was isolated from raw materials at levels media from 44.5% and from finished feed from 40.2%. In soybean and finished feed (sow and pregnant gilt) *A. candidus* was isolated with a frequency that varied from 10% to 21%. *Aspergillus oryzae* and *A. clavatus* were not isolated from raw materials alone but were isolated from pregnant girl and sow feed at low frequency. 

**Figure 3:**
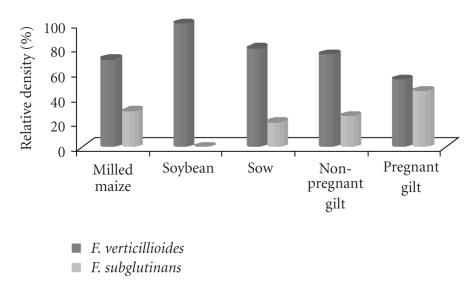
Relative density (%) of *Fusarium* spp. isolated from raw materials and finished feed for sow at different reproductive stages.

Two *Fusarium* spp. were identified. *F. verticillioides* was the predominant species in all analysed samples at levels that varied from 70% to 100% in raw materials and from 55% to 80% in finished feed. *Fusarium subglutinans* was only present in milled maize whereas this species was isolated from several kinds of finished samples at a relative density that varied from 20% to 45% (Figure 3).

**Figure 4:**
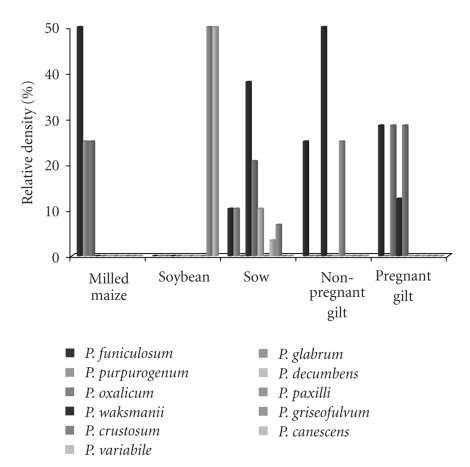
Relative density (%) of *Penicillium* spp. Isolated from raw materials and finished feed for sow at different reproductive stages.

Figure 4 shows the relative density of isolated* Penicillium* spp. from raw materials and finished feed. Eleven *Penicillium* spp. were isolated. In general, finished feed has higher *Penicillium* spp. diversity than that found in raw materials. *Penicillium funiculosum *was the prevalent isolated species, followed by *P. purpurogenum* and *P. oxalicum* in raw materials. In finished feed *P. funiculosum, P. waksmanii,* and *P. crustosum* were the prevalent isolated species.

Table 3 shows the AFB_1_, ZEA, and FB_1_ OTA levels found in raw materials and finished feed. Raw material samples did not show OTA, AFB_1_, and ZEA natural contamination levels. Milled maize (100%) and soybean samples (50%) were contaminated with FB_1_ at levels that varied from 50.3 to 1137.64 *μ*g Kg^−1^, respectively. None of the analyzed of raw material samples showed mycotoxin levels over the recommended limits for each studied mycotoxins (0.02 *μ*g g^−1^ for AFB_1_, 250 *μ*g kg^−1^ for OTA and 5000 *μ*g kg^−1^ for FBs). Among finished feed samples, only feed intended for sows showed AFB_1_ natural contamination levels at high frequency (80%). All Nonpregnant gilt samples were contaminated with low OTA levels whereas all of the other samples had not detected OTA levels or were below the quantification limit. All finished feed samples (100%) were contaminated with similar FB_1_ levels that ranged from 99.8 to 512.4 *μ*g Kg^−1^.

## 4. Discussion

Fungi and mycotoxins from raw materials and finished feed intended for sows at different reproductive stages were studied.

All analysed samples had counts over the proposed limits of 1 × 10^4^ CFU g^−1^ [20]. This result suggests a high fungal activity that could affect the palatability and reduce the nutrient adsorption [21, 22] determining a low hygienic quality and an improper storage. These results are similar to those obtained by other researchers in pig feed in Argentina [8, 10]. In this study, fungal species varied according to the analysed substrate. However, in general all samples showed that *Aspergillus* spp., *Penicillium* spp., and *Fusarium* spp., the main toxicogenic fungi, were the prevalent genera. *Aspergillus flavus* showed the highest relative density among *Aspergillus* spp. followed by *A. niger* aggregate spp. in raw materials. The same results were also found in finished feed. Several Brazilian authors obtained similar results to ours in maize and finished poultry and equine feed [23–25]. They found *A. flavus *as prevalent species followed by *A. candidus* and *A. niger* aggregate spp. Other studies from Argentina found *Aspergillus* section *Flavi* species as prevalent followed by *A. niger* aggregate spp. from poultry and pig feed and frequencies were similar to those found in Brazil [1, 26]. A high frequency of yeasts was also found. The significance of yeasts, which was frequently isolated, is not known. In this study, *F. verticillioides* was found as predominant followed by *F. subglutinans*. This prevalence has been informed from poultry feed in Latin America [23–27]. However, Argentinean samples always showed higher *F. verticillioides* isolation frequency than Brazilian samples. Pereyra et al. [10] obtained results similar to ours from finished pig feed samples in central Argentina. However, they did not report the fungal contamination present in raw materials and finished feed intended for sows. Several isolated *Penicillium* such as *P. oxalicum*, *P. raistrickii*, *P. paxilli*, *P. griseofulvum*, and *P. canescens *are involved in food spoilage and produce different toxic fungal metabolites (secalonic acid, griseofulvin, verruculogen, cyclopiazonic acid, penitrem A) [12]. There is little information regarding the toxicological effects of these mycotoxins in animals [2]. Home-cereals used in farms for feed elaboration probably provided fungal field contamination.

The prevailing field environmental conditions should be influencing initial FB_1_ and ZEA production by potential producers, whereas the inadequate storage conditions should be influencing the AFB_1_ and OTA production by potential producers, which were present in finished feed. Our previous studies carried out with FB_1_ and OTA feedstuff contamination demonstrated the absence of this toxin in raw materials but levels were detected in stored feed [8, 10].

It is important to state that the presence of fungi in feed does not automatically indicate the mycotoxin presence. In this work, the presence of *F. verticillioides* was related to the FB_1_ contamination. However, this behaviour was not shown by *A. flavus, A. niger *aggregate spp., *Fusarium* spp., and the presence of their mycotoxins in samples (AFs, OTA, and ZEA, resp.). Similar results were found in samples intended for pigs at different growth stages in central Argentina [10]. 

The present study has shown the simultaneous occurrence of carcinogenic mycotoxins. The cooccurrence of these toxins in pig feed was demonstrated in the central area of our country [8, 10]. However, none of these studies demonstrated the presence of fungi and mycotoxins in feed intended to pigs at reproductive stages. In animal production, the simultaneous occurrence of mycotoxins brings not only bad health in animals but also low production. Various mycotoxins may occur simultaneously depending on the environmental and substrate conditions [2]. Considering this coincident production, humans and animals may be exposed to mixtures rather than individual compounds. 

Regulations on standard products in the animal feed sector established that the current maximum permitted level for AFB_1_ for pigs is 0.02 *μ*g g^−1^ [24]. Aflatoxin B_1_ levels were higher than the recommended limits in 100% finished samples intended for sows. The European Union (EU) released guidance for OTA, FBs, and ZEA levels in animal feed (http://ec.europa.eu/information_society/activities/digital_libraries/doc/recommendation/recommendation/en.pdf) that establish allowed limits of 250 *μ*g kg^−1^ for OTA and 5000 *μ*g kg^−1^ for FBs. In our study, samples did not yield FBs and OTA levels higher than these allowed limits. 

The presence of mycotoxins indicates the existence of contamination. Since raw materials are a primary source of the moisture and fungi found in feed, the first important step in controlling moisture in feed is to control it in the raw materials from which the feed is prepared. Since all feed ingredients contain moisture, they should be monitored and their moisture content should be controlled in order to prevent the occurrence of mycotoxicoses in animal production, to reduce economic losses and to minimize hazards to human health.

This is the first study supplying data on fungi and the occurrence of mycotoxins in sow feed at different reproductive stages.

## Figures and Tables

**Table 1:** Composition (%) of feed intended for sows at different reproductive stages.

Ingredients	Reproduction sows		
	Sow	Nonpregnant gilt*	Pregnant gilt^†^
Milled maize	60	56	56
Deactivated soybean	4–25	30	26
Other cereals	0–10	2–6	10
Concentrated (sugar and vitamins mix)	5–15	2–8	0–8

*Feed used to increase ovulation from 20% to 30%.

^†^Feed intended to optimize the productive yields.

**Table 2:** Fungal counts (CFU/g) from raw materials and finished feed at different reproductive stages in DRBC and DG18 culture media.

Culture media	Sampling period	Fungal counts (CFU/g)* Media ± SD				
		Milled maize	Soybean	Sow	Nonpregnant gilt	Pregnant gilt
DRBC	May	6.7 × 10^4^ ± 4 × 10^3^	4.5 × 10^2^ ± 8 × 10^2^	1 × 10^4^ ± 5 × 10^3^	2 × 10^4^ ± 6 × 10^3^	5.9 × 10^4^ ± 4 × 10^3^
	June	9.7 × 10^4^ ± 5 × 10^3^	<1 × 10^2^	2 × 10^4^ ± 6 × 10^3^	5 × 10^4^ ± 7 × 10^3^	4 × 10^4^ ± 1 × 10^4^
	July	1.5 × 10^4^ ± 6 × 10^3^	<1 × 10^2^	2.2 × 10^4^ ± 7 × 10^3^	4 × 10^4^ ± 3 × 10^3^	4.1 × 10^4^ ± 4 × 10^3^
	August	2.3 × 10^5^ ± 7 × 10^5^	6 × 10^2^ ± 2 × 10^2^	1.9 × 10^4^ ± 1.4 × 10^3^	2.5 × 10^4^ ± 2.8 × 10^3^	3.9 × 10^4^ ± 4.2 × 10^3^
	September	2.9 × 10^5^ ± 9 × 10^3^	3 × 10^2^ ± 1 × 10^2^	1.4 × 10^5^ ± 5.6 × 10^4^	2.6 × 10^4^ ± 2.8 × 10^4^	2.3 × 10^5^ ± 2.8 × 10^4^

DG18	May	1.4 × 10^4^ ± 7 × 10^3^	8.5 × 10^2^ ± 9 × 10^2^	1 × 10^4^ ± 4 × 10^3^	1.5 × 10^4^ ± 3 × 10^3^	6.7 × 10^4^ ± 2.1 × 10^4^
	June	8 × 10^4^ ± 1.3 × 10^4^	<1 × 10^2^	1.5 × 10^4^ ± 5 × 10^3^	2 × 10^4^ ± 6 × 10^3^	1.3 × 10^4^ ± 5.3 × 10^4^
	July	1 × 10^4^ ± 9 × 10^3^	5 × 10^2^ ± 1 × 10^2^	1.7 × 10^4^ ± 6 × 10^3^	2.5 × 10^4^ ± 8 × 10^3^	5.4 × 10^4^ ± 4 × 10^3^
	August	1.3 × 10^5^ ± 2.1 × 10^4^	1 × 10^3^ ± 1 × 10^2^	2 × 10^4^ ± 2.1 × 10^3^	2.9 × 10^4^ ± 1.1 × 10^4^	3.8 × 10^4^ ± 3.5 × 10^3^
	September	1.5 × 10^5^ ± 2.5 × 10^4^	9 × 10^2^ ± 2 × 10^2^	5.9 × 10^5^ ± 4.9 × 10^3^	3.1 × 10^4^ ± 1.1 × 10^4^	3.9 × 10^5^ ± 7.8 × 10^4^

Detection limit: 1× 10^2^ CFU g^−1^. Maximum recommended level: 1 × 10^4^ CFU g^−1^ [16].

DRBC: dichloran rose bengal chloranphenicol. DG18: dichloran glycerol 18%.

**Table 3:** Mycotoxin levels found in raw materials and finished sow feed at different reproductive stages.

Mycotoxins		Raw material samples		Finished pig feed samples		
		Milled maize	Soybean	Sow	Nonpregnant gilt	Pregnant gilt
AFB_1_	Media ± SD* (*μ*g Kg^−1^)	ND^Ψ^	ND	228.2 ± 95	ND	ND
	Frequency (%)^†^	—	—	80	—	—
	Samples over limits (%)^‡^	—	—	100	—	—

OTA	Media ± SD (*μ*g Kg^−1^)	ND^Ψ^	ND	ND	0.259 ± 0.123	ND
	Frequency (%)	—	—	—	100	—
	Samples over limits (%)	—	—	—	0	—

FB_1_	Media ± SD (*μ*g Kg^−1^)	660.9 ± 415.7	82.8 ± 28.3	334.2 ± 178.4	353.1 ± 126.4	341.6 ± 118.2
	Frequency (%)	100	50	67	100	100
	Samples over limits (%)	0	0	0	0	0

ZEA	Media ± SD (*μ*g Kg^−1^)	ND	ND	ND	ND	ND
	Frequency (%)	—	—	—	—	—
	Samples over limits (%)	—	—	—	—	—

*SD: standard deviation of three replicates. ^†^Contamination frequency (%): percentage of samples contaminated with mycotoxin. ^‡^Percentage of samples contaminated with levels over the recommended: AFB_1_: 20 *μ*g Kg^−1^, OTA: 50 *μ*g Kg^−1^, ZEA: 100 *μ*g Kg^−1^ (pregnant), FB_1_: 5000 *μ*g Kg^−1^. ^Ψ^Not detected.
